# ZnT3 mRNA levels are reduced in Alzheimer's disease post-mortem brain

**DOI:** 10.1186/1750-1326-4-53

**Published:** 2009-12-23

**Authors:** Nancy Beyer, David TR Coulson, Shirley Heggarty, Rivka Ravid, G Brent Irvine, Jan Hellemans, Janet A Johnston

**Affiliations:** 1 Centre for Public Health, School of Medicine, Dentistry and Biomedical Sciences, Queen's University Belfast, Northern Ireland; 2 Genomics Core Facility, Queen's University Belfast, Regional Genetics Centre, Belfast City Hospital, Northern Ireland; 3 Brain Bank Consultants, Netherlands Institute for Neurosciences, Meibergdreef 47, 1105 BA Amsterdam, the Netherlands; 4 Center for Medical Genetics Ghent, Ghent University Hospital, B-9000 Ghent, Belgium

## Abstract

**Background:**

ZnT3 is a membrane Zn^2+ ^transporter that is responsible for concentrating Zn^2+ ^into neuronal presynaptic vesicles. Zn^2+ ^homeostasis in the brain is relevant to Alzheimer's disease (AD) because Zn^2+ ^released during neurotransmission may bind to Aβ peptides, accelerating the assembly of Aβ into oligomers which have been shown to impair synaptic function.

**Results:**

We quantified ZnT3 mRNA levels in Braak-staged human post mortem (pm) brain tissue from medial temporal gyrus, superior occipital gyrus, superior parietal gyrus, superior frontal gyrus and cerebellum from individuals with AD (n = 28), and matched controls (n = 5) using quantitative real-time PCR. ZnT3 mRNA levels were significantly decreased in all four cortical regions examined in the AD patients, to 45-60% of control levels. This reduction was already apparent at Braak stage 4 in most cortical regions examined. Quantification of neuronal and glial-specific markers in the same samples (neuron-specific enolase, NSE; and glial fibrillary acidic protein, GFAP) indicated that loss of cortical ZnT3 expression was more pronounced, and occurred prior to, significant loss of NSE expression in the tissue. Significant increases in cortical GFAP expression were apparent as the disease progressed. No gene expression changes were observed in the cerebellum, which is relatively spared of AD neuropathology.

**Conclusions:**

This first study to quantify ZnT3 mRNA levels in human pm brain tissue from individuals with AD and controls has revealed a significant loss of ZnT3 expression in cortical regions, suggesting that neuronal cells in particular show reduced expression of ZnT3 mRNA in the disease. This suggests that altered neuronal Zn^2+ ^handling may be an early event in AD pathogenesis.

## Background

ZnT3 is a Zn^2+ ^transporter which is a member of the solute carrier family 30 (SLC30), previously known as the cation diffusion facilitator (CDF) family. Studies in mice have shown that ZnT3 (SLC30A3) is primarily expressed in the brain, particularly in the hippocampus, amygdala and cerebral cortex [1]. Within neurons, ZnT3 protein was detected at membranes of small, round, clear synaptic vesicles from mossy fibre boutons in mouse and monkey hippocampus [1,2]. ZnT3 is responsible for concentrating Zn^2+ ^into these vesicles [3], which is co-released with glutamate upon depolarization [4,5]. Synaptic free Zn^2+ ^levels can reach high concentrations during neurotransmission (hundreds of μM) and its exact role at the synapse is unclear, although it has been shown to modulate postsynaptic events (both excitatory and inhibitory), probably via effects on GABA_A _[6] and NMDA receptors [7-9].

Several findings indicate that Zn^2+ ^homeostasis in the brain is of relevance in Alzheimer's disease (AD). Aβ peptides are found aggregated in amyloid plaques, one of the characteristic hallmarks of AD neuropathology. Aβ binds Zn^2+ ^and this rapid interaction accelerates Aβ aggregation on solid templates [10]. Current evidence indicates that lower order, soluble, Aβ assemblies (known as oligomers) are important in AD pathophysiology and that they impair synaptic function [11]. Zn^2+ ^released during neurotransmission was recently shown to play a key role in mediating formation of these Aβ oligomers at synapses [12]. Zn^2+^-induced Aβ aggregation can be prevented, and even reversed, with chelating agents [13]. Treatment of a transgenic mouse model relevant to AD (Tg2576) for three months with the metal chelating agent DP-109 reduced the appearance of amyloid plaques and increased soluble Aβ levels [14]. Crossing these animals with ZnT3 knock-out mice also resulted in reduced plaque load and the animals displayed markedly less cerebral amyloid angiopathy [15,16]. In patients with AD, Zn^2+ ^levels have been reported to be elevated in serum [17,18], and in brain tissue [19-21], particularly associated with amyloid plaques, cerebral amyloid angiopathy, and in cell bodies and dendrites of neurofibrillary tangle-positive neurons [22-24]. Compounds acting as metal ion chelators, such as clioquinol and PBT2, have entered clinical trials for AD. A slight improvement in clinical ratings was observed in twenty AD patients after a three week treatment with clioquinol [25], and it reduced plasma Aβ_42 _levels and increased Zn^2+ ^levels in a phase two clinical trial in AD [26].

These findings have prompted investigation of ZnT3 protein expression in the brains of another AD transgenic mouse model (APPswe/PS1dE9) and in AD patients. ZnT3 protein was abundantly detected in amyloid plaques throughout the cortex and hippocampus as well as amyloid angiopathic vessels in the APPswe/PS1dE9 mice, where levels were increased in comparison to wild-type littermates [27]. ZnT3 also co-localized with amyloid plaques throughout the cerebral cortex in five AD patients and was detected in vessels with amyloid angiopathy [28], although this study did not compare expression in control human brain tissue.

The present study is the first to investigate ZnT3 mRNA expression levels in human post mortem brains from individuals with AD, and matched controls. We used quantitative real-time PCR (RT qPCR), a fast, straightforward and reproducible technique which is increasingly becoming the method of choice for profiling mRNA levels due to its accuracy, wide dynamic range and sensitivity [29,30]. The accuracy of this technique is however totally dependent on the use of valid reference genes for data normalisation. Traditionally, 'housekeeping genes' such as glyceraldehyde 3-phosphate dehydrogenase (GAPDH) or β-actin have been used for normalisation, but if expression of these genes is altered by the disease process, then false negative or positive results may be obtained. It is therefore essential that the expression stability of the reference gene(s) is examined and confirmed in the appropriate disease state and tissue prior to using the gene for data normalisation. To identify a stable set of reference genes, we examined the expression stability of a range of candidate reference genes in post mortem brain samples from individuals with AD, Parkinson's disease, dementia with Lewy bodies, and controls [31]. The present study used this validated set of reference genes to investigate whether ZnT3 mRNA expression was altered in Braak-staged post mortem brain tissue from individuals with AD. Braak staging enabled the AD samples to be sub-grouped according to progression of the neurofibrillary tangle pathology [32]. We have also investigated the expression of a glial marker (glial fibrillary acidic protein, GFAP) and a neuronal marker (neuron-specific enolase, NSE) to investigate whether any observed variations in gene expression related to changing cell populations in the tissues sampled. We analysed five brain regions, affected to differing extents by AD neuropathology: cerebellum; medial temporal gyrus; superior occipital gyrus; superior parietal gyrus; and superior frontal gyrus.

This study identified significant reductions in cortical ZnT3 mRNA levels in AD, which occurred prior to overt loss of NSE expression, indicating that impaired neuronal Zn^2+ ^handling may be an early event in AD pathogenesis.

## Methods

### Post-mortem brain tissue samples

Human post mortem brain tissue was obtained from The Netherlands Brain Bank (Netherlands Institute for Neuroscience, Amsterdam, Netherlands), in accordance with local ethical approval, whereby autopsies on donors are performed only when a written consent from the donor or next of kin exists. The tissue originated from clinically well documented and neuropathologically confirmed cases and controls, with comprehensive information on ante- and post-mortem (pm) factors. All tissue was Braak staged [32]. Full details about the study subjects are presented in Table 1. 28 AD patients were included (9 at Braak stage 4; 11 at Braak stage 5; 8 at Braak stage 6) and 5 matched controls (4 at Braak stage 1; 1 at Braak stage 2). All individuals in the AD group had a clinical diagnosis of dementia or probable AD prior to death, and the neuropathological diagnosis was either AD, or AD/Lewy body variant (AD/LBV), as indicated on Table 1. The latter diagnosis was applied to brains where some cortical LB were observed, in addition to classical AD pathology. All controls also underwent a full neuropathological evaluation to confirm that they were not affected by AD or any other neurodegenerative disease. The tissue in this study was fresh-frozen after a generally short post-mortem delay and all tissue handling was done on dry ice to avoid thawing, which would lead to increased RNA degradation [33]. As shown in Table 1, there were no significant differences between the study groups in terms of post-mortem delay, previously reported to have a limited negative effect on brain mRNA levels [34]; or CSF pH, shown to have a major influence on brain mRNA levels [35,36]. Mean brain weight was significantly lower in the AD group, reflecting the brain atrophy caused by the disease. Frozen tissue blocks were obtained from cerebellum and four cortical gyri (medial temporal, superior occipital, superior parietal and superior frontal) of each individual and stored at -80°C.

**Table 1:** Information about the study subjects

Diagnosis	Gender	Age	Braak stage	pm delay (min)	CSF pH	Brain weight (g)
control	F	69	1	375	6.59	1264
control	F	72	1	405	6.52	1296
control	M	79	1	320	6.72	1322
control	M	83	1	275	6.49	1422
control	F	88	2	340	n.d.	1152

**Control mean**	**3F/2 M**	**78.2**	**1.2**	**343**	**6.58**	**1291**

AD	F	84	4	230	6.64	1196
AD	F	85	4	220	6.59	1065
AD	F	93	4	335	6.65	1194
AD	F	84	4	230	6.64	1196
AD	F	87	4	240	6.91	955
AD	F	101	4	265	6.93	1016
AD	M	91	4	250	n.d.	1160
AD/LBV	F	81	4	193	6.54	1096
AD/LBV	F	76	4	295	6.78	1180

AD	M	67	5	260	6.62	1094
AD	F	77	5	252	6.43	1151
AD	F	77	5	215	6.67	1235
AD	M	86	5	335	6.39	1315
AD	M	95	5	185	6.4	1203
AD	M	70	5	525	6.25	1220
AD/LBV	M	80	5	1670	6.19	1170
AD/LBV	F	69	5	295	6.59	1039
AD/LBV	F	84	5	365	6.1	1116
AD/LBV	F	87	5	260	6.17	1022
AD/LBV	M	84	5	275	6.54	1373

AD	M	58	6	385	6.42	1273
AD	F	87	6	300	6.66	852
AD	F	87	6	250	6.9	1047
AD	F	77	6	285	6.74	1116
AD	F	91	6	275	6.55	949
AD	F	66	6	215	6.42	1105
AD/LBV	M	54	6	610	6.51	1334
AD/LBV	F	69	6	295	6.41	1003

**AD mean**	**19 F/9 M**	**80.3**	**5**	**340**	**6.54**	**1131**

There were no statistically significant differences in age, cerebrospinal fluid (CSF) pH or pm delay between AD patients (Braak stages 4, 5 and 6) and controls (Braak stage 1 and 2). Brain weight was significantly lower in the AD patients, compared to controls (p = 0.01, unpaired t-test).

### Extraction of total RNA and cDNA synthesis

Tissue samples of approximately 100 mg were cut from fresh-frozen post-mortem brain tissue blocks on dry ice, and incubated with QIAzol lysis reagent (Qiagen). Samples were homogenised using a TissueLyser (Qiagen) and total RNA was extracted using the RNeasy^® ^lipid tissue mini kit (Qiagen). To study RNA integrity, total RNA preparations from cerebellum and medial temporal gyrus of six AD patients and five controls were analysed using an Agilent 2100 Bioanalyzer (Agilent Technologies) in a previous study in our laboratory (described in full by [31]). 2 μl of total RNA from each sample were used for spectrophotometric quantitation using NanoDrop^® ^ND-1000 (NanoDrop^®^). 2 μg RNA were reverse transcribed using the Omniscript^® ^reverse transcription kit (Qiagen) according to the manufacturer's protocol, in 20 μl reactions containing 0.5 μM oligo-dT and 5 μM random nonomer primers (Operon).

### Real-time PCR

qPCR reactions were carried out with cDNA-specific TaqMan^® ^gene expression assays using an ABI 7900HT real-time PCR system (Applied Biosystems). Further information about the gene expression assays is presented in Table 2. Each reaction contained 2 μl cDNA (corresponding to 20 ng reverse transcribed RNA), 0.25 μl TaqMan^® ^gene expression assay for the relevant gene, 2.5 μl TaqMan^® ^universal PCR master mix (Applied Biosystems) and 0.25 μl RNAse free water in a total volume of 5 μl. Sealed 384 well plates were subjected to the following thermal cycling conditions: 95°C for 10 min, followed by 40 cycles of 95°C for 15 sec and 60°C for 1 min. The reactions were performed in triplicate and each qPCR run contained a non-template (negative) control and two inter-run calibrators.

**Table 2:** TaqMan^® ^assay identification (ID) numbers, amplicon length and probe binding sequence

Gene name	Assay ID	Probe Binding Sequence (5'-3')	Amplicon length
ZnT3	Hs00185728_m1	CAC CTG GCC ATC GAC TCC ACC GCT G	93
SDHA	Hs00417200_m1	CGC CGC CGT GGT CGA GCT AGA AAA T	124
UBC	Hs00824723_m1	GTG ATC GTC ACT TGA CAA TGC AGA T	71
B2 M	Hs99999907_m1	TTA AGT GGG ATC GAG ACA TGT AAG C	75

### PCR Efficiency

**Figure 1:**
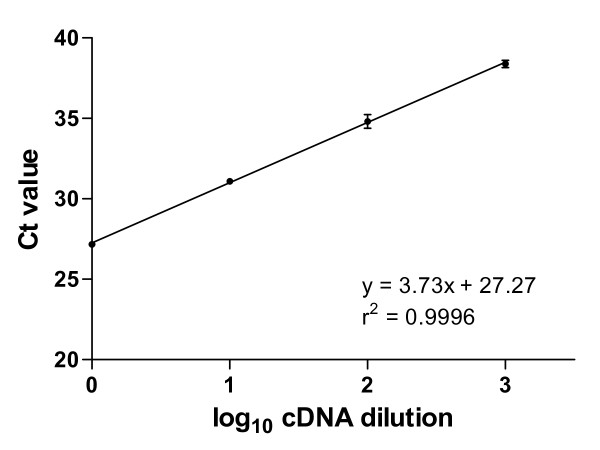
**ZnT3 efficiency plot**. Quadruplicate ZnT3 qPCR analyses were carried out using the indicated dilutions of a cDNA pool, derived as described in the Methods section. Mean Ct values ± SD are presented for each dilution where the assay signal rose above the detection threshold. PCR reaction efficiency for the ZnT3 TaqMan assay (85%) was calculated from the slope of the line as described in the Methods section.

A pool of cDNAs prepared from four different brain regions of seven AD patients and two controls was used to generate PCR efficiency curves. 10-fold serial dilutions of the cDNA pool, ranging from ×1 dilution to ×1000000, were used in quadruplicate qPCR reactions, carried out as described above. Mean cycle threshold (Ct) values for each cDNA dilution were plotted against the log_10 _of the cDNA input, generating an efficiency plot (Figure 1). Reaction efficiency (E) for each assay was calculated using the slope of this line in the following equation: E = 10^(-1/slope) ^-1.

### Data Analysis

Raw Ct values generated by the TaqMan^® ^gene expression assays were imported into the qBase Plus (Biogazelle) software [15]. The qBase Plus algorithm enables normalisation to more than one reference gene and is able to take into account gene-specific E, which distinguishes it from the ^ΔΔ^Ct method [37]. In addition, it also employs the signals from the inter-run calibrators to minimize run-to-run variation [38]. In this study, ubiquitin C (UBC), succinate dehydrogenase complex-subunit A (SDHA) and beta-2-microglobulin (B2M) were analysed as potential reference genes, as their expression levels were demonstrated to be sufficiently stable in human pm brain tissue affected by neurodegenerative disease in our previous study [31]. During the data normalisation process in qBase Plus it was clear that reference gene stability decreased considerably when B2M was included. We therefore used UBC and SDHA to normalise the ZnT3 data presented.

### Statistics

Statistical analysis was performed using GraphPad Prism version 4.00 or InStat version 3.05 for Windows; GraphPad Software, San Diego California USA, http://www.graphpad.com. Non-parametric statistical tests were used where data were not normally distributed, or where there were significant differences between group standard deviations.

## Results

### PCR efficiency

A pool of cDNA from four brain regions of AD patients and controls was generated and 10-fold serial dilutions assayed in order to determine PCR reaction efficiency from the slope of the log_10 _cDNA vs Ct plot. PCR reaction efficiency for the ZnT3 gene expression assay was 85% (Figure 1). ZnT3 gene expression was normalised using the two reference genes UBC and SDHA, whose efficiencies were previously determined to be 99% and 95%, respectively, in a separate study using the same cDNA pool in our laboratory [31]. The efficiency plots for all three TaqMan^® ^gene expression assays had r^2 ^values of ≥ 0.996.

### ZnT3 expression in pm brain tissue

**Figure 2:**
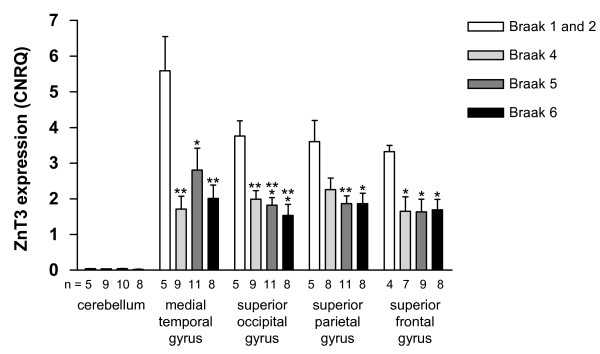
**ZnT3 expression in AD and control brain tissue**. RNA was isolated from the pm brain tissue regions indicated and reverse transcribed prior to qPCR analysis in triplicate, and data analysis using qBase Plus, as described in the Methods section. Columns represent the mean ZnT3 expression level (expressed as CNRQ) ± SEM for each Braak stage, and the numbers at the base of each column (n) indicate the number of individuals included in that analysis. Statistical analysis (one-way ANOVA and Tukey-Kramer multiple comparisons test) revealed significant differences between control ZnT3 expression levels (Braak stages 1 and 2), and ZnT3 expression levels in the AD brain tissue (Braak stages 4, 5, and 6): * p < 0.05; ** p < 0.01; *** p < 0.001 compared to control ZnT3 expression in the same brain region.

We analysed the gene expression level of ZnT3 in 28 AD patients and 5 age-matched controls (Table 1). Comparison of all AD patients (Braak 4, 5 and 6) with all controls (Braak 1 and 2) showed that ZnT3 expression was significantly decreased in all four cortical regions examined in the AD patients, compared to controls. ZnT3 mRNA levels were reduced by 60% in the medial temporal gyrus (p = 0.0002, unpaired t-test); 52% in superior occipital gyrus (p < 0.0001, unpaired t-test); 48% in superior frontal gyrus (p = 0.002, unpaired t-test) and by 45% in the superior parietal gyrus (p = 0.009, Mann-Whitney). In the cerebellum, ZnT3 expression level was extremely low (less than 1% of the cortical expression levels in control samples) and no significant differences were detectable between the study groups (20% lower in AD group, p = 0.55, unpaired t-test). Figure 2 presents these data in more detail, sub-grouped according to Braak stage. The statistically significant reductions in ZnT3 expression level were already present by Braak stage 4 in all cortical regions examined, except for the superior parietal gyrus where the reduction did not reach significance until Braak stage 5 (one-way ANOVA and Tukey-Kramer multiple comparisons test).

### Expression of neuronal and glial-specific genes in pm brain tissue

We determined the mRNA expression levels of NSE, as a neuronal-specific marker, and GFAP, as a glial-specific marker, in these pm brain tissue samples in a separate study (unpublished data). This identified significant changes in expression of these genes as the disease progressed (as indicated by Braak stage). NSE expression was negatively correlated with Braak stage (Spearman correlation, p < 0.05) in medial temporal gyrus and superior parietal gyrus, indicating significant loss of neuronal gene expression in these regions as the disease progressed. This correlation just failed to reach statistical significance in the superior frontal gyrus (Spearman correlation, p = 0.056). Pooling of all AD patients (Braak 4, 5 and 6), for comparison with all controls (Braak 1 and 2), showed that NSE expression was significantly decreased in superior frontal gyrus in AD (44% reduction), while the decreases in NSE mRNA levels in superior parietal gyrus (27% reduction), superior occipital gyrus (17% reduction) and medial temporal gyrus (9% reduction) did not reach statistical significance. Kruskall-Wallis comparison of NSE mRNA levels in the individual Braak goups 4, 5, or 6 versus controls (Braak 1 and 2), did not reveal any statistically significant changes.

In contrast, GFAP expression showed significant positive correlations with Braak stage in medial temporal gyrus, superior occipital gyrus, superior parietal gyrus and superior frontal gyrus (Spearman correlation, p < 0.05), indicating significant gliosis as the disease progressed. Pooling of all AD patients (Braak 4, 5 and 6), for comparison with all controls (Braak 1 and 2), showed that GFAP expression was significantly increased in medial temporal gyrus affected by AD (to 364% of control, p = 0.01 Mann-Whitney); in superior occipital gyrus (391% of control, p = 0.01 Mann-Whitney); and superior frontal gyrus (330% of control, p = 0.04 Mann-Whitney); but this increase just failed to achieve statistical significance in superior parietal gyrus (173% of control, p = 0.09 Mann-Whitney). Kruskall-Wallis comparison of GFAP mRNA levels in the individual Braak goups 4, 5, or 6 versus controls (Braak 1 and 2), showed statistically significant increases at Braak stage 6 in medial temporal gyrus, superior occipital gyrus and superior parietal gyrus.

Notably, no statistically significant changes in NSE or GFAP expression were present in the cerebellum, a region which is relatively spared of AD-related neuropathology.

### Ratio of ZnT3 expression to neuronal and glial-specific gene expression

The methodology employed in this study did not allow visualisation of the cellular source of the ZnT3 mRNA detected. As a first step towards exploring whether the changes we observed in ZnT3 mRNA expression were linked to the changing cell populations in the AD cortical tissue, we calculated the ratio of ZnT3 expression (CNRQ) to NSE, and to GFAP CNRQs for each individual and brain region. We reasoned that if ZnT3 expression was purely neuronal, then the ZnT3/NSE ratio in each individual sample would be predicted to remain constant over the course of the disease (as indicated by Braak stage).

**Figure 3:**
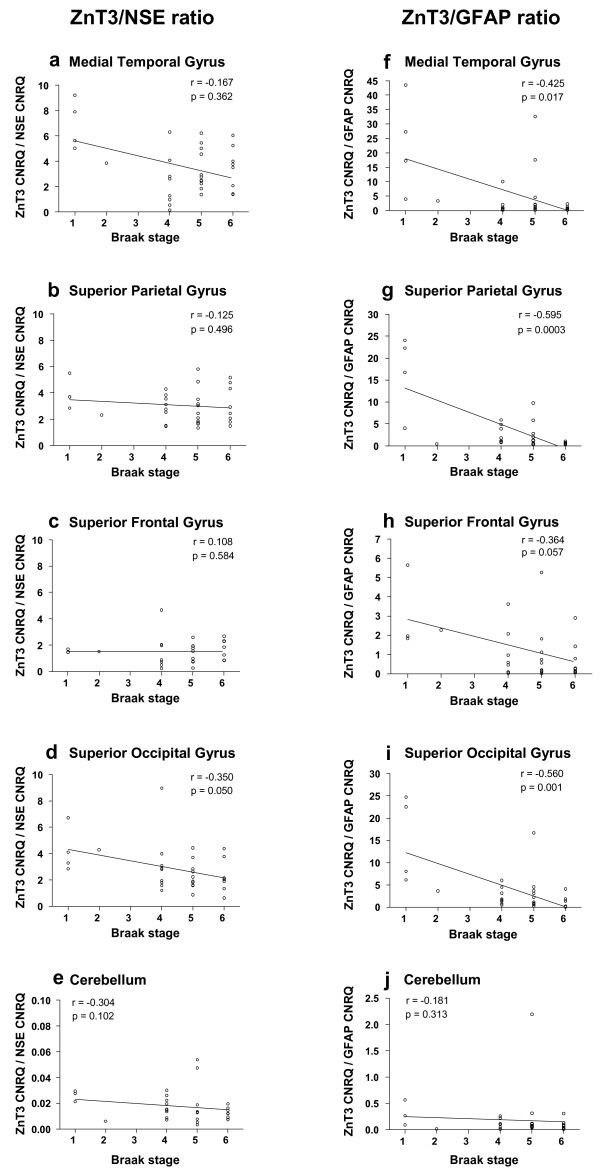
**ZnT3/NSE and ZnT3/GFAP CNRQ in AD and control brain tissue**. ZnT3 mean CNRQ values were divided by either mean NSE CNRQ, or mean GFAP CNRQ, for the same sample. This produced the ratios, which are shown plotted against the relevant Braak stage for that sample. Spearman correlation coefficients (r) are displayed on each graph, alongside the relevant p value.

In Figure 3 (a, b, c, d, e), it can be seen that there was a decrease in the ZnT3 CNRQ/NSE CNRQ ratio in the superior occipital gyrus as the disease progressed which achieved borderline statistical significance, but no significant correlations between this ratio and Braak stage in any of the other brain regions investigated. There was however a consistent negative correlation between the ZnT3 CNRQ/GFAP CNRQ and Braak stage which was statistically significant in the medial temporal gyrus, superior parietal gyrus and superior occipital gyrus, and was close to statistical significance in the superior frontal gyrus (Figure 3f, g, h, i, j). These findings appear to confirm that ZnT3 expression was predominantly neuronal, since the ratio between ZnT3 and NSE expression remained relatively constant, despite the significant losses in NSE expression observed as the disease progressed in cortical regions. In addition, the increased levels of GFAP were not accompanied by increased levels of ZnT3 (negative correlation), indicating that ZnT3 may not be expressed by glial cells.

In the cerebellum, neither ratio showed any significant correlation with Braak stage.

## Discussion

This study identified a significant loss of ZnT3 mRNA expression in four cortical regions in AD which occurred early in the course of the disease (Figure 2). The reductions in cortical ZnT3 gene expression, by 45-60%, were already well-established at Braak stage 4, when neocortical regions are still relatively unaffected by AD-related neurofibrillary tangle pathology and overt neuronal loss [32]. To study this directly in our samples, we analysed expression of a neuronal marker (NSE) in this Braak-staged tissue, which revealed significantly reduced NSE expression as the disease progressed, reflecting the loss of neuronal cells found in the disease. At Braak stage 4, however, we detected no significant changes in NSE expression in any cortical region. This indicates that the changes in ZnT3 expression occurred before significant neuronal losses had occurred. We provide indirect evidence that pm brain ZnT3 expression was predominantly neuronal (Figure 3), although our methodological approach did not allow direct identification of the cellular source of ZnT3 mRNA. The fact that the ZnT3/NSE ratio did not generally correlate with Braak stage indicates that expression of these two mRNAs was decreasing roughly in parallel, even though the ZnT3 changes were apparent earlier than NSE changes as discussed above. In addition, we identified increased GFAP expression in the AD pm brain tissue (cortical regions), reflecting the astrogliosis and astrocyte activation associated with the disease, as reported by others [39,40]. However, the ZnT3/GFAP ratio showed a negative correlation with Braak stage, indicating that the gliosis at later stages of the disease was not associated with increased ZnT3 expression. This suggests that glia were not a major source of the ZnT3 mRNA detected here. This is consistent with what is already known about the localization of ZnT3 protein, in neuronal presynaptic vesicle membranes [1,2]. The very low expression level we detected in the cerebellum was also consistent with previous reports in mice [1,3]. Taken together, the present study suggests that cortical neuronal cells show reduced expression of ZnT3 mRNA in brain tissue affected by AD, before NSE expression is compromised. This suggests that the loss of ZnT3 expression is not solely due to the ongoing neuronal cell death in AD, although this undoubtedly contributes to it at later stages. One potential explanation for this finding is that whilst NSE is expressed in most neurons, Znt3 expression is more localised, as has been shown in mouse and monkey [2]. Hence, the loss of ZnT3 expression prior to loss of NSE could reflect differences in susceptibility of ZnT3-expressing neurons to AD.

This is the first direct study of ZnT3 mRNA expression in human pm brain affected by AD to our knowledge. There has been one previous study of ZnT3 protein levels in pm brain in AD, which detected ZnT3 in the vicinity of amyloid plaques and blood vessels with amyloid angiopathy [28], although no control subjects were studied. Increased protein levels of other zinc transporters in the same family, which move zinc across different membranes to ZnT3 (ZnT-1, ZnT-4 and ZnT-6), have been previously reported in AD and mild cognitive impairment [41]. Our finding of decreased ZnT3 mRNA expression in the disease is perhaps unexpected, and would not have been predicted by previous studies of ZnT3 expression in rodents. These studies would tend to predict that increased ZnT3 mRNA expression may be associated with AD, while decreased expression may be protective. For example, ZnT3 gene knock-out in an AD transgenic mouse model (Tg2576) resulted in reduced plaque load and cerebral amyloid angiopathy [15,16], and a study of another AD transgenic mouse model (APPswe/PS1dE9) detected increased ZnT3 protein levels in comparison to wild-type littermates [27]. Previous work investigating regulation of ZnT3 expression has demonstrated a role for estrogen. Ovariectomy raised ZnT3 levels and hippocampal synaptic vesicle Zn^2+ ^levels, while estrogen replacement lowered these [42]. These changes were attributed to estrogen-induced reductions in expression of the delta subunit of trans-Golgi network adaptor-like complex AP-3. Genetic ablation of the delta subunit of AP-3 in mocha mice leads to reduced cortical vesicular ZnT3 protein expression and Zn^2+ ^levels [43]. Given that early menopause and low estrogen levels have been linked to increased risk for AD, these findings suggest that increased ZnT3 levels may be associated with AD. However this area is not clear-cut and while human epidemiological data suggest that hormone replacement therapy (HRT) reduces AD risk, some experimental evidence demonstrates that HRT can increase the incidence of dementia [44]. Dietary omega-3 polyunsaturated fatty acid has also been shown to regulate ZnT3 expression. Rats supplied with an omega-3-deficient diet perinatally had increased expression of ZnT3 and increased levels of hippocampal Zn^2+ ^in adulthood [45]. Taken together with the reported decrease in this class of fatty acids in pm brains from AD patients, this again predicts that increased ZnT3 expression may be associated with AD [46]. However, one study of a mouse model of accelerated aging (senescence-accelerated mouse prone 10, SAMP10) identified reduced ZnT3 expression as the mice aged. This was associated with increased expression of GFAP and excessive hippocampal glutamatergic excitotoxicity, with deterioration of learning and memory in the mice [47]. This corresponds more closely to the changes we observed in the AD pm brain tissue, where ZnT3 expression was decreased, and GFAP increased.

## Conclusions

The present study indicates that ZnT3 mRNA levels are significantly reduced in the cortex in AD. The finding that this change occurs prior to overt loss of NSE expression indicates that it may precede wide-spread neuronal cell loss. The mechanism(s) underlying this change require further investigation to determine whether loss of ZnT3 expression exacerbates the disease, or occurs as a protective mechanism to reduce synaptic Zn^2+ ^levels in the face of AD-related increases in brain Zn^2+ ^levels. Either way, this finding indicates that altered neuronal Zn^2+ ^handling may be an early event in AD pathogenesis.

## Competing interests

The authors declare that they have no competing interests.

## Authors' contributions

NB designed and carried out the qPCR experiments, analysed data and drafted the manuscript. DTRC prepared RNA and the majority of the cDNA stocks and carried out NSE and GFAP qPCR analyses. SH contributed to the design and execution of the qPCR analyses. RR provided the pm brain tissue and commented on neuropathological aspects of the manuscript. GBI participated in the design and coordination of the study. JH contributed to data analysis. JAJ coordinated the study, finalised data analyses and contributed substantially to writing the manuscript. All authors read and approved the final manuscript.
